# Supplementary material for: Nigral volume loss in prodromal, early, and moderate Parkinson’s disease
Source: NPJ Parkinsons Dis. 2025 Jun 21;11:181. doi: 10.1038/s41531-025-00976-3 (PMC12182591; doi:10.1038/s41531-025-00976-3)
Supplement: Supplementary file 1 — Supplementary Material [file 41531_2025_976_MOESM1_ESM.pdf]

PPMI STUDY TEAMS/CORES/COLLABORATORS FOR PUBLICATIONS

Executive Steering Committee:
Kenneth Marek, MD[5] (Principal Investigator); Caroline Tanner, MD, PhD[6]; Tanya Simuni, MD[7]; Andrew Siderowf, MD, MSCE[8]; Douglas Galasko, MD[9]; Lana Chahine, MD[10]; Christopher Coffey, PhD[11]; Kalpana Merchant, PhD[12]; Kathleen Poston, MD[13]; Roseanne Dobkin, PhD[14]; Tatiana Foroud, PhD[15]; Brit Mollenhauer, MD[16]; Dan Weintraub, MD[8]; Ethan Brown, MD[6]; Karl Kieburtz, MD, MPH[17]; Mark Frasier, PhD[18]; Todd Sherer, PhD[18]; Sohini Chowdhury, MA[18]; Roy Alcalay, MD[19] and Aleksandar Videnovic, MD[20]

Steering Committee:
Duygu Tosun-Turgut, PhD[6]; Werner Poewe, MD[21]; Susan Bressman, MD[22]; Jan Hammer[15]; Raymond James, RN[23]; Ekemini Riley, PhD[24]; John Seibyl, MD[5]; Leslie Shaw, PhD[8]; David Standaert, MD, PhD[25]; Sneha Mantri, MD, MS[26]; Nabila Dahodwala, MD[8]; Michael Schwarzschild[20]; Connie Marras[27]; Hubert Fernandez, MD[28]; Ira Shoulson, MD[17]; Helen Rowbotha[29]; Paola Casalin[30] and Claudia Trenkwalder, MD[16]

Michael J. Fox Foundation (Sponsor): Todd Sherer, PhD; Sohini Chowdhury, MA; Mark Frasier, PhD; Jamie Eberling, PhD; Katie Kopil, PhD; Alyssa O'Grady; Maggie McGuire Kuhl; Leslie Kirsch, EdD and Tawny Willson, MBS

Study Cores, Committees and Related Studies: ***(Include as applicable to the paper)***
Project Management Core: Emily Flagg, BA[5]
Site Management Core: Tanya Simuni, MD[7]; Bridget McMahon, BS[5]
Strategy and Technical Operations: Craig Stanley, PhD[5]; Kim Fabrizio, BA[5]
Data Management Core: Dixie Ecklund, MBA, MSN[11]; Trevis Huff, BSE[11]
Screening Core: Tatiana Foroud, PhD[15]; Laura Heathers, BA[15]; Christopher Hobbick, BSCE[15]; Gena Antonopoulos, BSN[15]
Imaging Core: John Seibyl, MD[5]; Kathleen Poston, MD[13]
Statistics Core: Christopher Coffey, PhD[11]; Chelsea Caspell-Garcia, MS[11]; Michael Brumm, MS[11]
Bioinformatics Core: Arthur Toga, PhD[31]; Karen Crawford, MLIS[31] *Biorepository Core:* Tatiana Foroud, PhD[15]; Jan Hamer, BS[15]
Biologics Review Committee: Brit Mollenhauer[16]; Doug Galasko[9]; Kalpana Merchant[12]
Genetics Core: Andrew Singleton, PhD[32]
Pathology Core: Tatiana Foroud, PhD[15]; Thomas Montine, MD, PhD[13]
Found: Caroline Tanner, MD PhD[6]
PPMI Online: Carlie Tanner, MD PhD[6]; Ethan Brown, MD[6]; Lana Chahine, MD[10]; Roseann Dobkin, PhD[14]; Monica Korell, MPH[6]

Site Investigators:
Charles Adler, PhD[33]; Roy Alcalay, MD[19]; Amy Amara, PhD[34]; Paolo Barone, PhD[35]; Bastiaan Bloem, PhD[36]; Susan Bressman, MD[22]; Kathrin Brockmann, MD[37]; Norbert Brüggemann, MD[38]; Lana Chahine, MD[10]; Kelvin Chou, MD[39]; Nabila Dahodwala, MD[8]; Alberto Espay, MD[40]; Stewart Factor, DO[41]; Hubert Fernandez, MD[28]; Michelle Fullard, MD[34]; Douglas Galasko, MD[9]; Robert Hauser, MD[42]; Penelope Hogarth, MD[43]; Shu-Ching Hu, PhD[44]; Michele Hu, PhD[45]; Stuart Isaacson, MD[46]; Christine Klein, MD[38]; Rejko Krueger, MD[29]; Mark Lew, MD[47]; Zoltan Mari, MD[48]; Connie Marras, PhD[27]; Maria Jose Martí, PhD[49]; Nikolaus McFarland, PhD[50]; Tiago Mestre, PhD[51]; Brit Mollenhauer, MD[16]; Emile Moukheiber, MD[52]; Alastair Noyce, PhD[53]; Wolfgang Oertel, PhD[54]; Njideka Okubadejo, MD[55]; Sarah O'Shea, MD[56]; Rajesh Pahwa, MD[57]; Nicola Pavese, PhD[58]; Werner Poewe, MD[21]; Ron Postuma, MD[59]; Giulietta Riboldi, MD[60]; Lauren Ruffrage, MS[25]; Javier Ruiz Martinez, PhD[61]; David Russell, PhD[5]; Marie H Saint-Hilaire, MD[23]; Neil Santos, BS[33]; Wesley Schlett[20]; Ruth Schneider, MD[17]; Holly Shill, MD[62]; David Shprecher, DO[63]; Tanya Simuni, MD[7]; David Standaert, PhD[25]; Leonidas Stefanis, PhD[64]; Yen Tai, PhD[65]; Caroline Tanner, PhD[6]; Arjun Tarakad, MD[66]; Eduardo Tolosa PhD[49] and Aleksandar Videnovic, MD[20]

Coordinators:
Susan Ainscough, BA[35]; Courtney Blair, MA[25]; Erica Botting[42]; Isabella Chung, BS[48]; Kelly Clark[63]; Ioana Croitoru[61]; Kelly DeLano, MS[40]; Iris Egner, PhD[21]; Fahrial Esha, BS[60]; May Eshel, MSc[19]; Frank Ferrari, BS[39]; Victoria Kate Foster[58]; Alicia Garrido, MD[49]; Madita Grümmer[38]; Bethzaida Herrera[62]; Ella Hilt[37]; Chloe Huntzinger, BA[34]; Raymond James, BS[23]; Farah Kausar, PhD[6]; Christos Koros, MD, PhD[64]; Yara Krasowski, MSc[36]; Dustin Le, BS[43]; Ying Liu, MD[34]; Taina M. Marques, PhD[29]; Helen Mejia Santana, MA[56]; Sherri Mosovsky, MPH[10]; Jennifer Mule, BS[28]; Philip Ng, BS[27]; Lauren O'Brien[57]; Abiola Ogunleye, PGDip[65]; Oluwadamilola Ojo, MD[55]; Obi Onyinanya, BS[52]; Lisbeth Pennente, BA[46]; Romina Perrotti[59]; Michael Pileggi, MS[59]; Ashwini Ramachandran, MSc[8]; Deborah Raymond, MS[22]; Jamil Razzaque, MS[45]; Shawna Reddie, BA[51]; Kori Ribb, BSN[52]; Kyle Rizer, BA[50]; Janelle Rodriguez, BS[9]; Stephanie Roman, HS[5]; Clarissa Sanchez, MPH[66]; Cristina Simonet, PhD[65]; Anisha Singh, BS[17]; Elisabeth Sittig, RN[54]; Barbara Sommerfeld MSN[41]; Angela Stovall, BS[39]; Bobbie Stubbeman, BS[40]; Alejandra Valenzuela, BS[47]; Catherine Wandell, BS[44]; Diana Willeke[16]; Karen Williams, BA[7] and Dilinuer Wubuli, MB[27]

Partners Scientific Advisory Board (Acknowledgement)

Funding: PPMI – a public-private partnership – is funded by the Michael J. Fox Foundation for Parkinson's Research and funding partners, including 4D Pharma, Abbvie, AcureX, Allergan, Amathus Therapeutics, Aligning Science Across Parkinson's, AskBio, Avid Radiopharmaceuticals, BIAL, BioArctic, Biogen, Biohaven, BioLegend, BlueRock Therapeutics, Bristol-Myers Squibb, Calico Labs, Capsida Biotherapeutics, Celgene, Cerevel Therapeutics, Coave Therapeutics, DaCapo Brainscience, Denali, Edmond J. Safra Foundation, Eli Lilly, Gain Therapeutics, GE HealthCare, Genentech, GSK, Golub Capital, Handl Therapeutics, Insitro, Jazz

Pharmaceuticals, Johnson & Johnson Innovative Medicine, Lundbeck, Merck, Meso Scale Discovery, Mission Therapeutics, Neurocrine Biosciences, Neuron23, Neuropore, Pfizer, Piramal, Prevail Therapeutics, Roche, Sanofi, Servier, Sun Pharma Advanced Research Company, Takeda, Teva, UCB, Vanqua Bio, Verily, Voyager Therapeutics, the Weston Family Foundation and Yumanity Therapeutics.
5 Institute for Neurodegenerative Disorders, New Haven, CT
6 University of California, San Francisco, CA
7 Northwestern University, Chicago, IL
8 University of Pennsylvania, Philadelphia, PA
9 University of California, San Diego, CA
10 University of Pittsburgh, Pittsburgh, PA
11 University of Iowa, Iowa City, IA
12 TransThera Consulting
13 Stanford University, Stanford, CA
14 Rutgers University, Robert Wood Johnson Medical School, New Brunswick, New Jersey
15 Indiana University, Indianapolis, IN
16 Paracelsus-Elena Klinik, Kassel, Germany
17 University of Rochester, Rochester, NY
18 The Michael J. Fox Foundation for Parkinson's Research, New York, NY
19 Tel Aviv Sourasky Medical Center, Tel Aviv, Israel
20 Massachusetts General Hospital, Boston, MA
21 Innsbruck Medical University, Innsbruck, Austria
22 Mount Sinai Beth Israel, New York, NY
23 Boston University, Boston, MA
24 Center for Strategy Philanthropy at Milken Institute, Washington D.C.
25 University of Alabama at Birmingham, Birmingham, AL
26 Duke University, Durham, NC
27 Toronto Western Hospital, Toronto, Canada
28 Cleveland Clinic, Cleveland, OH
29 University of Luxembourg, Luxembourg
30 BioRep, Milan, Italy
31 Laboratory of Neuroimaging (LONI), University of Southern California
32 National Institute on Aging, NIH, Bethesda, MD
33 Mayo Clinic Arizona, Scottsdale, AZ
34 University of Colorado, Aurora, CO
35 University of Salerno, Salerno, Italy
36 Radboud University, Nijmegen, Netherlands
37 University of Tübingen, Tübingen, Germany
38 Universität Lübeck, Luebeck, Germany
39 University of Michigan, Ann Arbor, MI
40 University of Cincinnati, Cincinnati, OH
41 Emory University of Medicine, Atlanta, GA
42 University of South Florida, Tampa, FL
43 Oregon Health and Science University, Portland, OR
44 University of Washington, Seattle, WA
45 John Radcliffe Hospital Oxford and Oxford University, Oxford, UK
46 Parkinson's Disease and Movement Disorders Center, Boca Raton, FL
47 University of Southern California, Los Angeles, CA
48 Cleveland Clinic-Las Vegas Lou Ruvo Center for Brain Health, Las Vegas, NV
49 Hospital Clinic of Barcelona, Barcelona, Spain
50 University of Florida, Gainesville, FL
51 The Ottawa Hospital, Ottawa, Canada
52 Johns Hopkins University, Baltimore, MD
53 Wolfson Institute of Population Health, Queen Mary University of London, UK
54 Philipps-University Marburg, Germany
55 University of Lagos, Nigeria
56 Columbia University Irving Medical Center, New York, NY
57 University of Kansas Medical Center, Kansas City, KS
58 Clinical Ageing Research Unit, Newcastle, UK

59 Montreal Neurological Institute and Hospital/McGill, Montreal, QC, Canada
60 NYU Langone Medical Center, New York, NY
61 Hospital Universitario Donostia, San Sebastian, Spain
62 Barrow Neurological Institute, Phoenix, AZ
63 Banner Research Institute, Sun City, AZ
64 National and Kapodistrian University of Athens, Athens, Greece
65 Imperial College of London, London, UK
66 Baylor College of Medicine, Houston, TX
